# Primary observations of EVO ICL implantation for high myopia with concave iris

**DOI:** 10.1186/s40662-023-00335-4

**Published:** 2023-04-02

**Authors:** Zhe Zhang, Lingling Niu, Tingting Liu, Yang Shen, Jianmin Shang, Jing Zhao, Ruoyan Wei, Xingtao Zhou, Peijun Yao

**Affiliations:** 1 Department of Ophthalmology and Vision Science, Eye and ENT Hospital, Fudan University, 19 Baoqing Road, Xuhui District, Shanghai, China; 2 NHC Key Laboratory of Myopia, Fudan University, Shanghai, China; 3 Laboratory of Myopia, Chinese Academy of Medical Sciences, Shanghai, China

**Keywords:** EVO ICL, High myopia, Concave iris, Ultrasound biomicroscope, Pigment dispersion

## Abstract

**Purpose:**

To investigate the morphological changes of concave iris in myopic patients after EVO implantable collamer lens (ICL) implantation.

**Methods:**

EVO ICL candidates with posterior bowing iris were observed using ultrasound biometric microscopy (UBM) in this prospective nonrandomized observational study. Forty patients were enrolled, with 20 patients in the concave iris group and the other 20 patients in the control group. None of the patients underwent laser peripheral iridotomy. All patients received preoperative and postoperative examinations, which included uncorrected distance visual acuity (UDVA), corrected distance visual acuity (CDVA), subjective manifest refraction and intraocular pressure. UBM was used to observe iris curvature (IC), irido-corneal angle (ICA), posterior chamber angle (PCA), iris-lens contact distance (ILCD), iris-zonule distance (IZD) and ciliary process length (CPL). Anterior chamber angle pigment was observed by gonioscopy. The preoperative and postoperative data were analyzed using SPSS.

**Results:**

The average follow-up period was 13.3 ± 5.3 months. The mean efficacy indices were 1.10 ± 0.13 and 1.07 ± 0.11 (*P* = 0.58), and the safety indices were 1.19 ± 0.09 and 1.18 ± 0.17 in the control group and the concave iris group *(P* = 0.93), respectively. The IOP postoperatively were 14.13 ± 2.02 mmHg and 14.69 ± 1.59 mmHg in control and concave iris groups (*P* = 0.37). Preoperatively, the concave iris group was presented with greater IC (*P* < 0.0001), longer ILCD (*P* < 0.0001), wider ICA (*P* = 0.004), narrower PCA (*P* = 0.01), and shorter IZD (*P* = 0.03) than the control group. In the concave iris group, IC, ILCD and ICA were significantly decreased after ICL implantation (*P* < 0.0001), while PCA and IZD were significantly increased (*P* = 0.03 and *P* = 0.04, respectively). Postoperative IC, ILCD, ICA, PCA and IZD were not statistically different between groups (*P* > 0.05). There was no significant difference in pigment deposition grades between the two groups (*P* = 0.37).

**Conclusion:**

After EVO ICL implantation, the morphology of concave iris was significantly improved, which may reduce the risk of intraocular pigment dissemination caused by iris concavity. The concave iris has no impact on the safety of EVO ICL surgery during the follow-up.

**Supplementary Information:**

The online version contains supplementary material available at 10.1186/s40662-023-00335-4.

## Background

The prevalence of myopia has markedly increased in Asia, and consequences of myopia included some of the most common causes of irreversible blindness and socioeconomic burden. Since approved by the FDA in 2005, the implantation of implantable collamer lens (ICL™, STAAR Surgical, Nidau, Switzerland), a posterior chamber phakic intraocular lens, has proven to be safe and effective to correct high myopia [1–3]. ICL implantation affords many advantages over other refractive surgeries on treating high myopia, including retaining accommodative ability of crystalline lens and avoiding irreversible damage to relatively healthy corneal tissue. Compared to moderate and mild myopia, high myopia may benefit from ICL in the case of inadequate corneal thickness or abnormal corneal morphology [4, 5].

High myopia is more likely to be accompanied by structural abnormalities of the anterior chamber, such as deep anterior chamber depth and concave iris [6]. Concave iris is a typical characteristic of pigment dispersion syndrome [7]. Posterior bowing of the midperipheral iris can result in contact with the lens zonules and then causes pigment shedding [8]. Over time, the increase in pigment can lead to the impairment of the outflow facility in the trabecular meshwork with an elevation in intraocular pressure (IOP) [9]. Pigment dispersion syndrome leads to pigmentary glaucoma when the pigment granules accumulate in the eye's drainage system. Reverse pupil block is believed to be the main cause of the concave iris configuration [8, 10]. The initial treatment consists of laser peripheral iridotomy to remove any components contributing to pupillary blockage [11].

The EVO ICL is based on V4 generation but has a 360 μm central hole. This “Central FLOW Technology” design can regulate the compliance of aqueous humor flow between the ICL and the crystalline lens to eliminate the need for preoperative laser peripheral iridotomy [12]. The central hole can connect the anterior and posterior chambers and allow more natural aqueous humor circulation. Various studies have reported good refractive outcomes with the EVO ICL in eyes with moderate to high myopia [13]. These eyes also maintained good IOP control and did not develop pupillary block glaucoma and cataract [14–17]. Kawamorita et al. simulated the dynamics of aqueous humor after EVO ICL implantation, suggesting that the central-hole ICL improves circulation of the aqueous humor on the anterior surface of the crystalline lens [18]. Therefore, we hypothesized that the concave iris morphology would improve after implantation of EVO ICL without a preoperative laser peripheral iridotomy.

Here, we observed morphological changes of the concave iris using ultrasound biomicroscopy (UBM) and the evaluated position and safety of EVO ICL in high myopes with concave irises.

## Methods

### Study design

In this observational study, forty patients with an average age of 28.53 ± 4.06 years were enrolled. Twenty patients were assigned to the concave iris group and the other 20 patients were in the control group. All surgeries were performed by one surgeon (XZ) from June 2019 to November 2020, and during that time, we had a total of 8078 patients underwent EVO ICL implantation, so the frequency of patients presenting concave iris morphology was about 20/8078. The average follow-up period was 13.3 ± 5.3 months. Table 1 outlines the baseline data of all patients.

**Table 1:** Biometric data of subjects at baseline

Characteristic	Control group	Concave iris group	*P* value
Gender (M/F)	6/14	5/15	0.8
Age (years)	28.60 ± 3.60	28.45 ± 4.57	0.91
SER (D)	− 10.86 ± 3.39	− 12.13 ± 2.13	0.17
IOP (mmHg)	14.46 ± 1.95	14.31 ± 2.47	0.84
ACD (mm)	3.11 ± 0.28	3.29 ± 0.43	0.16
AL (mm)	29.18 ± 1.85	29.81 ± 2.00	0.33
ICL size (13.7/13.2/12.6)	4/8/8	4/6/10	0.78

*M* = male; *F* =female; *SER* = spherical equivalent refraction; *IOP* = intraocular pressure; *ACD* = anterior chamber distance; *AL* = axial length; *ICL* = implantable collamer lens

**Fig. 1:**
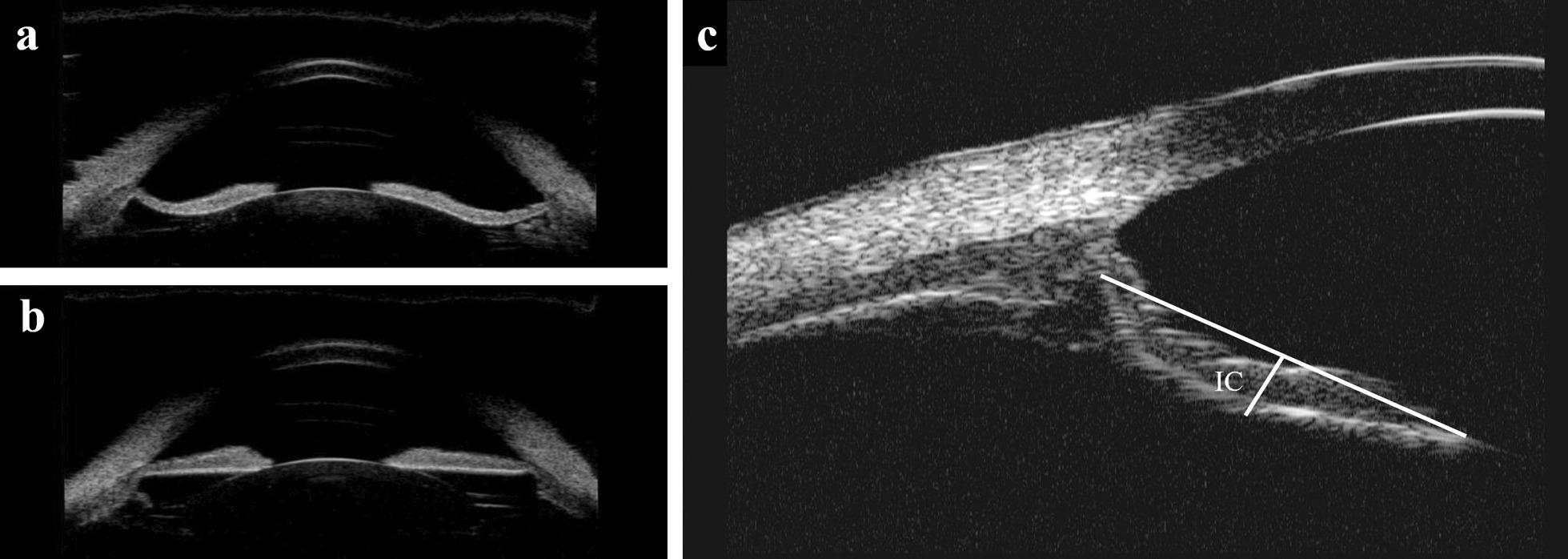
Description of iris morphology. **a** Typical concave iris in the concave iris group; **b** Typical planar iris in the control group; **c** Measurement of iris curvature (IC)

UBM was used to analyze the iris curvature (IC) and categorize different iris shapes [19] (Fig. 1a and b). Iris curvature was determined as the extent of posterior bowing of the iris and is measured by drawing a line from the iris root to the point of the pupil margin and then measuring the maximum perpendicular distance from the drawn line to the iris pigment epithelium [20] (Fig. 1c). The value of the iris curvature was considered positive if the iris showed a posterior deflection while "zero" or negative values were considered as belonging to the control group [21]. UBM was used to obtain the anterior segment parameters at baseline and follow-up visits.

This observational study was approved by the Fudan University EENT Hospital Review Board (No. 2016038), and all work was carried out in accordance with the Declaration of Helsinki. All patients were fully informed of the details and potential risks of the procedure, and written informed consent was obtained.

The inclusion criteria were age 20 to 42 years, stable refractive error (≤ 0.50 D change per year in refractive error in the past two years), minimum anterior chamber depth of 2.8 mm and a minimum endothelial cell density (ECD) of 2000 cells/mm^2^, no contact lens use for at least two weeks. The exclusion criteria were the presence of comorbid eye disorders, suspicion of keratectasia and presence of comorbid systemic diseases.

### Examination

#### Clinical examination

All patients underwent preoperative and postoperative ocular examinations. The following main parameters were evaluated: uncorrected distance visual acuity (UDVA), corrected distance visual acuity (CDVA), subjective manifest refraction, IOP (Canon, Japan), corneal topography and vault (Pentacam HR, Oculus Optikgeräte GmbH, Wetzlar, Germany) and UBM (Quantel medical, French). Anterior chamber angle pigment was observed by gonioscopy according to the five-class system developed by Scheie [22].

#### UBM measurement

**Fig. 2:**
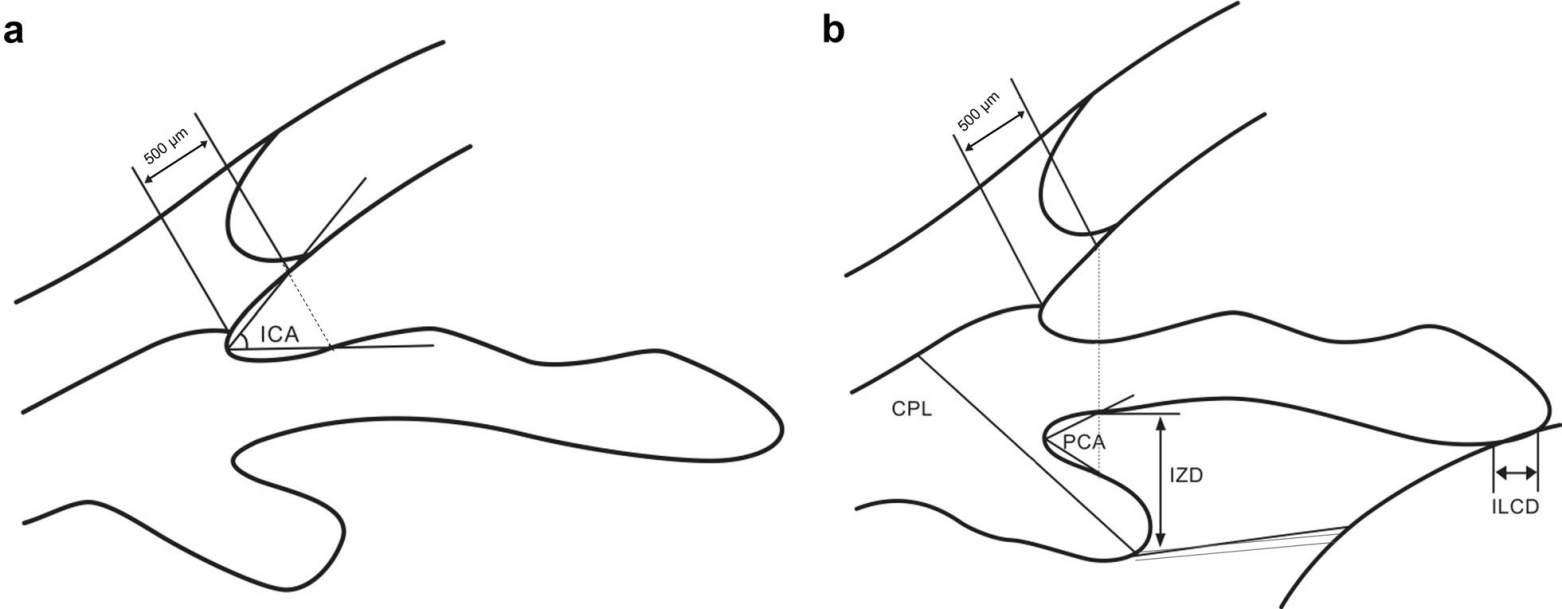
Ultrasound biomicroscopy (UBM) images of an eye showing the measurement of: **a** anterior chamber: irido-corneal angle (ICA), **b** posterior chamber: iris-lens contact distance (ILCD), posterior chamber angle (PCA), iris-zonule distance (IZD), ciliary process length (CPL)

All eyes were examined with the UBM. The patient was lying supine under standard room illumination, fixating on a target at a distance. All examinations were done by the same operator (LLN) and related parameters measured by another operator (ZZ). Topical 0.4% oxybuprocaine was instilled and an appropriately sized eye cup was inserted between the lids and filled with 2.5% methylcellulose and saline. The transducer tip was then placed in the fluid and each eye was examined at the 3-, 6-, 9-, and 12-o’clock positions. The following parameters (Fig. 2) were assessed using the linear and angular caliper provided by the instrument software, taking the average of the four directions and measuring to the second decimal position [23, 24].

Iris-lens/iris-ICL contact distance (ILCD): Determined by measuring the distance along the iris pigmented epithelium from the pupillary border to the point where the anterior lens (or EVO ICL) surface leaves the iris.

Anterior chamber angle (irido-corneal angle, ICA): Measured with the apex in the iris recess, and the arms of the angle passing through a point on the trabecular meshwork at 500 μm from the scleral spur and the point on the iris perpendicularly opposite. The size of the anterior chamber angle is given in degrees.

Posterior chamber angle (PCA): Measured with the apex in the posterior chamber recess, and the arms of the angle passing through the posterior border of iris and anterior border of the ciliary body at two points where a line extends from the corneal endothelium at 500 μm from the scleral spur. The size of the posterior chamber angle is given in degrees.

Posterior chamber distance (iris-zonule distance, IZD): This corresponds to the posterior chamber depth measured from the posterior iris surface (iris pigmented epithelium) to the first visible zonular fiber at a point just clearing the ciliary process.

Ciliary process length (CPL): Determined as the longest length in a straight line between the apex and the base of the ciliary process, as close as possible to a perpendicular from the sclera.

### Surgical procedure

The surgical technique of ICL V4c implantations has described previously [25]. Briefly, pupils were dilated preoperatively. EVO ICL was implanted via a 3 mm temporal corneal incision by an injector cartridge. Then, the EVO ICL (STAAR Surgical, Nidau, Switzerland) was placed in the posterior chamber. After the surgery, a topical antibiotic (0.5% levofloxacin, Cravit, Santen, Osaka, Japan) was administered four times per day for seven days. A topical steroid (1.0% prednisolone acetate, Pred Forte; Allergan, Irvine, CA, USA) was used four times daily for four days, pranoprofen (Senju, Osaka, Japan) was used four times daily for 14 days and Natriumhyaluronat (Hycosan, Germany) was used four times daily for three months.

### EVO implantable collamer lens

The power calculation of the EVO ICL was performed using a modified vertex formula based on the preoperative refractive parameters, according to the manufacturer’s instructions. The size of the EVO ICL was determined from the white-to-white and anterior chamber depth both obtained by Pentacam HR (Oculus Optikgeräte GmbH, Wetzlar, Germany).

### Statistical analyses

Only data from the right eyes were included for analysis. All statistical analyses were performed using SPSS version 23 (IBM Corp, USA). The data were presented as the mean ± standard deviation. Normality of data was assessed using the Shapiro–Wilk test, and data were normal in all cases. The baseline biometric data and variables including iris curvature, ILCD, anterior and posterior chamber angle, distance of posterior chamber and ciliary process length were compared using unpaired *t*-test. Wilcoxon signed rank test was used to evaluate the pigment amount of the two groups. A *P* value of less than 0.05 was considered statistically significant.

## Results

### Efficacy and safety of EVO ICL implantation

**Fig. 3:**
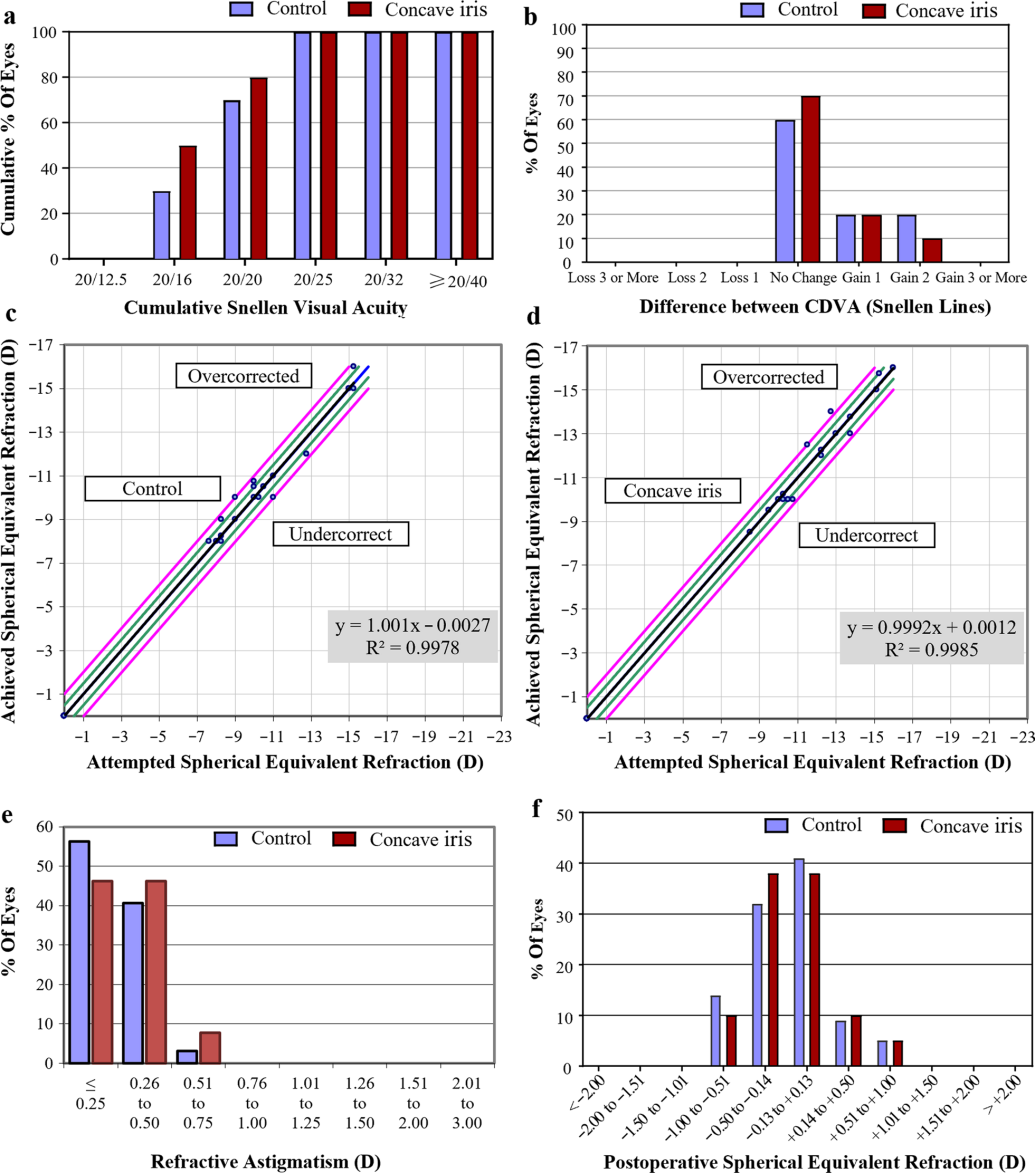
Refractive outcomes after implantable collamer lens (ICL) in the control and concave iris groups. **a** Cumulative uncorrected distance visual acuity (UDVA); **b** Postoperative UDVA versus preoperative corrected distance visual acuity (CDVA); Attempted versus achieved spherical equivalent (SE) refraction after ICL in the control group (**c**) and concave iris group (**d**); **e** Refractive astigmatism; **f** SE refraction

All procedures were successful. No vision-threatening complications occurred during follow-up. The mean efficacy indices (postoperative UDVA/preoperative CDVA) were 1.10 ± 0.13 and 1.07 ± 0.11 in the control group and concave iris group, respectively (*P* = 0.58). The safety indices (postoperative CDVA/preoperative CDVA) were 1.19 ± 0.09 and 1.18 ± 0.17 in the control group and concave iris group, respectively (*P* = 0.93). No eyes from either group lost one or more lines of CDVA (Fig. 3). In the control group, the ECD changed from 2810 ± 356 cells/mm^2^ preoperatively to 2718 ± 298 cells/mm^2^ postoperatively (*P* = 0.42). In the concave iris group, the ECD changed from 2892 ± 475 cells/mm^2^ preoperatively to 2714 ± 342 cells/mm^2^ postoperatively (*P* = 0.22). The postoperative IOP were 14.13 ± 2.02 mmHg and 14.69 ± 1.59 mmHg in the control and concave iris groups (*P* = 0.37), respectively. There was no significant difference in ECD between groups before and after the operation. The vault of the control group and concave iris group was 582 ± 177 μm and 588 ± 209 μm, respectively (*P* = 0.93).

### Changes in iris curvature

**Fig. 4:**
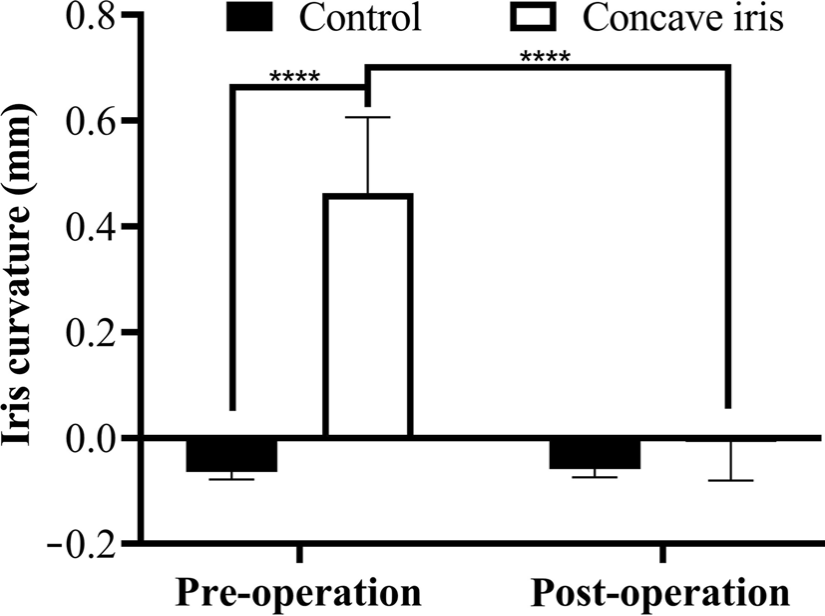
Iris curvature of the control group and concave iris group. ****P < 0.0001

The preoperative IC of the concave iris group was significantly greater than that of the control group (*P* < 0.0001). In the concave iris group, the IC reduced (*P* < 0.0001), and was not different from that of the control group (*P* = 0.07; Fig. 4).

### Changes in iris–lens/iris-ICL contact distance

**Fig. 5:**
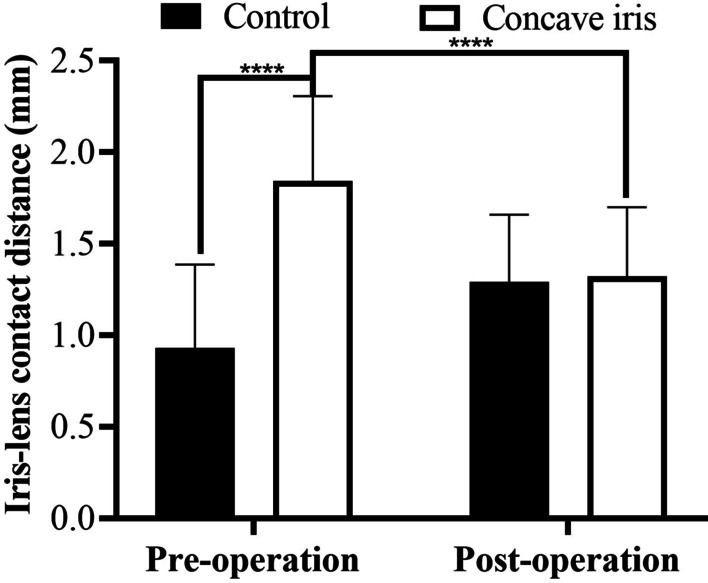
Iris-lens contact distance of the control group and concave iris group. ****P < 0.0001

The preoperative ILCD of the concave iris group was significantly longer than that of the control group (*P* < 0.0001). During the postoperative follow-up, the ILCD of the concave iris group was significantly reduced (*P* < 0.0001), but not different from the ILCD of the control group (*P* = 0.80; Fig. 5).

### Changes in the morphology of the anterior and posterior chambers

The CPL of the concave iris group was 0.61 ± 0.29 mm, which is not significantly different from that of the control group (0.63 ± 0.17 mm, *P* = 0.78), preoperatively. Furthermore, preoperatively, the ICA was wider and the PCA was narrower in the concave iris group than in the control group. In the control group, the ICA reduced after surgery, but PCA did not change significantly. In the concave iris group, the ICA significantly reduced after surgery while the PCA increased. There was no significant difference in ICA and PCA between the two groups postoperatively (Table 2).

**Table 2:** Chamber angle changes in two groups

Parameter	Anterior chamber angle (ICA) (°)			Posterior chamber angle (PCA) (°)		
	Control	Concave iris	*P* value	Control	Concave iris	*P* value
Preoperative	39.62 ± 9.66	55.10 ± 14.98	0.004	83.90 ± 28.93	60.97 ± 19.90	0.01
Postoperative	30.42 ± 9.72	28.61 ± 8.74	0.52	75.40 ± 30.38	79.66 ± 26.84	0.70
*P* value	0.009	< 0.0001	–	0.46	0.03	–

*ICA* = irido-corneal angle

**Fig. 6:**
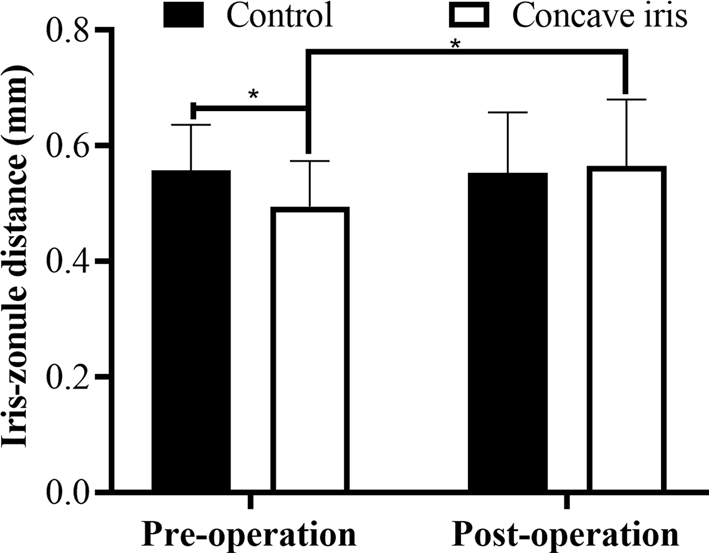
Posterior chamber distance of the control group and concave iris group. *P < 0.05.

The IZD of the concave iris group was significantly shorter than that of the control group preoperatively (*P* = 0.03). During the postoperative follow-up, the IZD of the concave iris group increased, but not significantly different from the IZD of the control group (*P* = 0.80; Fig. 6).

### Anterior chamber angle pigment

Observed with gonioscopy, the pigment amount of grades 0, 1 and 2 were found to be 45% (9/20), 50% (10/20), 5% (1/20) in the control group and 30% (6/20), 65% (13/20), 5% (1/20) in the concave iris group postoperatively (*P* = 0.61; Additional file 1: Fig. S1).

## Discussion

This study was meant to evaluate the morphological changes after EVO ICL implantation in concave iris eyes compared with normal eyes. EVO ICL is a safe and effective option for the surgical correction of high myopia. In our study, all cases in both groups gained good postoperative visual acuity and also showed no serious complications, indicating that concave iris had no direct impact on the visual outcome in ICL surgery.

With the development of UBM, quantitative examination of the anterior segment and the iris profile became possible [26]. UBM has significantly contributed to the understanding of the anatomic variations of concave iris [23]. In order to make correct comparisons, we selected a control group matched for age and refraction with the concave iris eyes being studied. This study identified anatomical features, as measured by UBM, capable of differentiating eyes with concave iris from normal controls. In concave iris eyes, posterior bowing of the peripheral iris can be observed, while the iris is plane or slightly convex in a normal eye. The ILCD was also analyzed, proving to be significantly longer in concave iris eyes than in normal. The posterior chamber of concave iris eyes is more crowded than that of normal eyes. These anatomical configuration in concave iris eyes could be considered a result of reverse pupillary block.

Reverse pupillary block is thought to be the pathophysiologic mechanism of concave iris eyes that increases the extent of ILCD and prevents pressure equalization between the anterior and posterior chambers [27]. This leads to the pressure in the anterior chamber being greater than in the posterior chamber, resulting in the posterior bowing of the iris. Moreover, a concave iris can cause pigment shedding from iridozonular contact and the development of pigment dispersion. The circulating pigment settling in the trabecular meshwork leads to an increased IOP [28].

Our study found that the anatomic factors of concave iris eyes were alleviated after ICL implantation, and there was no significant difference in IC between the concave iris and control groups postoperatively. The ILCD of concave iris eyes was also reduced to normal eye levels after ICL implantation. IZD was increased in the concave iris eyes postoperatively, which indicated that the distance between the iris and zonular increased and that the iris is no longer in contact with the zonular. The increased values of IZD and PCA also indicated that EVO ICL implantation relieved the congestion in the posterior chamber. At the same time, there was no increased risk of anterior chamber crowding.

Concave iris patients changed from a posterior curvature to a planar configuration partly due to ICL's contact with the posterior surface of the iris, giving it a physical support. On the other hand, all the patients in this study were implanted with EVO ICL. The central hole offers a channel for aqueous humor to circulate between the anterior and posterior chambers, and thus provides a stable IOP [29]. This might be the main reason why the planar morphology of the iris was regained after EVO ICL implantation. The central hole seems to play a similar role to peripheral iridotomy, removing pupillary blocks [30] and improving the shape of concave irises.

Campbell et al. proposed that contact between zonular fibers and the iris posterior pigment epithelium resulted in pigment dispersion in concave iris patients [31]. The EVO ICL implant eliminates contact between anterior zonular fibers and the iris posterior pigment epithelium, reduces constant rubbing of the iris, and eliminates the occurrence of pigment dispersion. Although there was still contact between the iris and ICL, we observed a significant reduction in iris-ICL contact in patients with concave irises. Considering the high biocompatibility of the ICL, the contact between the iris and the ICL, the soft, elastic, and hydrophilic surface is similar to the anterior capsule of the natural lens and may prevent a mechanical loss of pigment [3]. The mean pigment amounts in the anterior chamber angle were within normal limits, without abnormal pigment amount of grades 3 and 4 being found, in 80 eyes postoperatively. IOP was stable throughout the follow-up visit in both groups in this study. In the concave iris group, none of our cases developed secondary glaucoma following excessive vault or pigment dispersion during the follow-up since the friction between the lens and the iris was diminished.

There also was no significant difference in ciliary body length. Previous studies had defined an excellent vault to be from 250 to 750 μm [32]. In our study, we found that the postoperative mean vault had no statistical difference between two groups. The central vault is mainly affected by the size of EVO ICL and the sulcus-to-sulcus diameter, which would explain why concave irises had no direct impact on the change of central vault.

There are some limitations to this study. First, this study had a short follow-up period. Second, the sample size was small. However, this study proved the improvement in morphology of concave iris in patients after EVO ICL implantation. Based on our findings, EVO ICL implantation is safe and effective in these concave iris patients.

## Conclusion

After EVO ICL implantation, the morphology of the concave iris was significantly improved, which may reduce the risk of intraocular pigment dissemination caused by iris concavity.

## Supplementary Information

**Additional file 1: Figure S1.** Case presentation showing concave iris after implantable collamer lens (ICL) implantation. A representative case (22-year-old male) is presented. The patient’s preoperative iris curvature (IC), iris-lens contact distance (ILCD), irido-corneal angle (ICA), posterior chamber angle (PCA) and iris-zonule distance (IZD) were 0.66 mm, 2.18 mm, 84.98°, 53°, and 0.38 mm, respectively. At 8 months after surgery, IC, ILCD, ICA, PCA and IZD were 0.06 mm, 1.25 mm, 27.30°, 88.50°, 0.56 mm, respectively. The vault was 660 µm. No obvious pigmentation was observed in the gonioscopy 8 months after surgery. UBM images before and after ICL implantation are shown (**a, b** preoperative; **c, d** 8 months after surgery). **e** Gonioscopy at 8 months postoperatively.

## Data Availability

The data that support the findings of this study are available from the corresponding author upon reasonable request.
