# Supplementary figures and images for: Primary observations of EVO ICL implantation for high myopia with concave iris
Source: Eye Vis (Lond). 2023 Apr 2;10:18. doi: 10.1186/s40662-023-00335-4 (PMC10068169; doi:10.1186/s40662-023-00335-4)

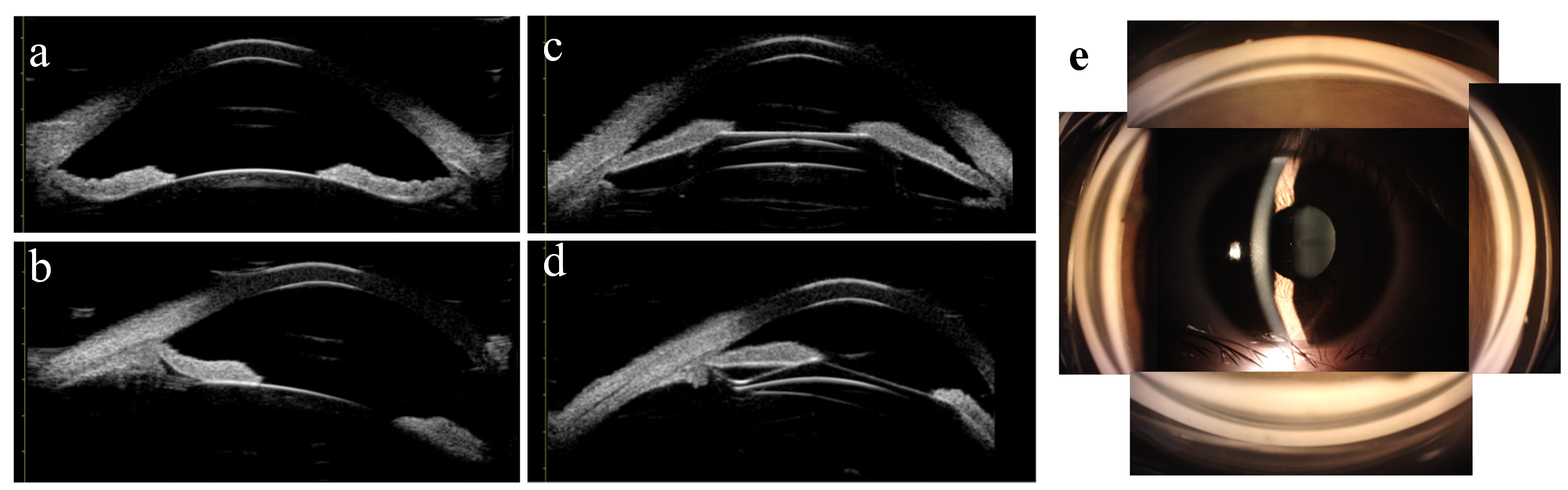

Supplement: Supplementary file 1 — Additional file 1: Figure S1. Case presentation showing concave iris after implantable collamer lens (ICL) implantation. A representative case (22-year-old male) is presented. The patient’s preoperative iris curvature (IC), iris-lens contact distance (ILCD), irido-corneal angle (ICA), posterior chamber angle (PCA) and iris-zonule distance (IZD) were 0.66 mm, 2.18 mm, 84.98°, 53°, and 0.38 mm, respectively. At 8 months after surgery, IC, ILCD, ICA, PCA and IZD were 0.06 mm, 1.25 mm, 27.30°, 88.50°, 0.56 mm, respectively. The vault was 660 µm. No obvious pigmentation was observed in the gonioscopy 8 months after surgery. UBM images before and after ICL implantation are shown (a, b preoperative; c, d 8 months after surgery). e Gonioscopy at 8 months postoperatively. [file 40662_2023_335_MOESM1_ESM.tif]
